# ERH Interacts With EIF2α and Regulates the EIF2α/ATF4/CHOP Pathway in Bladder Cancer Cells

**DOI:** 10.3389/fonc.2022.871687

**Published:** 2022-06-14

**Authors:** Kun Pang, Yang Dong, Lin Hao, Zhen-duo Shi, Zhi-guo Zhang, Bo Chen, Harry Feng, Yu-yang Ma, Hao Xu, Deng Pan, Zhe-sheng Chen, Cong-hui Han

**Affiliations:** ^1^Department of Urology, Xuzhou Central Hospital, Xuzhou Clinical School of Xuzhou Medical College, Jiangsu, China; ^2^STEM Academic Department, Wyoming Seminary, Kinston, PA, United States; ^3^Graduate School, Bengbu Medical College, Bengbu, China; ^4^College of Pharmacy and Health Sciences, St. John’s University, Queens, NY, United States

**Keywords:** ERH protein, bladder cancer (BC), protein–protein interaction, EIF2α, EIF2α-ATF4/CHOP pathway

## Abstract

**Background:**

There is a lack of research on the molecular interaction of the enhancers of rudimentary homolog (ERH) in bladder cancer (BC) cells. This study aimed to determine the interacting proteins of ERH in human T24 cells.

**Methods:**

First, the *ERH* gene was overexpressed in human T24 cells. Coimmunoprecipitation (co-IP) and shotgun mass spectrometry (MS) analyses were performed to obtain a list of proteins that interact with ERH. Subsequently, bioinformatic analyses with Gene Ontology (GO), Kyoto Encyclopedia of Genes and Genomes (KEGG) and protein–protein interaction (PPI) studies were performed to analyze the ERH-interactive protein list (ERH-IPL). Then, we selected one of the interacting proteins, EIF2α for verification. An immunofluorescence colocalization assay was performed to validate the co-expression of the selected protein, and the binding sites of the two proteins were predicted by ZDOCK technology. Finally, PCR analysis on the downstream molecules of the interacting protein was performed for verification.

**Results:**

ERH protein was successfully overexpressed in human T24 cells. We obtained a list of 205 proteins that might directly or indirectly interact with the ERH protein by mass spectrometric analysis. The bioinformatic analysis showed that ERH-interacting proteins were related to “ribonucleoprotein complex”, “ATPase activity”, “nuclear speck”, and “translation factor activity, RNA binding”. We further identified one of the key genes, EIF2S1, and confirmed that the corresponding protein EIF2α is co-expressed and may bind with ERH in human T24 cells. The mRNA levels of molecules ATF4 and CHOP were found to be upregulated by ERH.

**Conclusion:**

ERH protein affects “ribonucleoprotein complex”, “ATPase activity”, “nuclear speck”, and “translation factor activity, RNA binding”. The ERH protein can interact with EIF2α and regulate the EIF2α-ATF4/CHOP signaling pathway in human T24 cells.

## 1 Introduction

Bladder carcinoma (BC) is the fourth most common cancer and the second leading cancer in the male genitourinary system (1). BC resulted in 12,260 deaths in 2020 and ranked the 8^th^ in male malignant tumors in the United States (2). Accumulating studies have revealed that the enhancers of the rudimentary homolog (ERH) are involved in the occurrence and development of malignant tumors (3, 4). In a previous study, we found that the ERH gene can affect the migration and invasion of human T24 cells by regulating the expression of MYC (5). In addition, we found at the genetic level that ERH may inhibit apoptosis through regulating TLR, NF-κB, TNF or TGF-beta signaling pathways in human T24 cells to ultimately promote the growth of malignant tumors (6). However, gene expression profiling results cannot predict or confirm the cellular role of the ERH protein. Although the upstream and downstream regulation of ERH in bladder cancer cells had been demonstrated, there are few reports on the ERH -interacting proteins in bladder cancer cells.

The interaction of ERH protein with a specific protein or subunit could affect the biological effects of ERH (7–10). Therefore, a comprehensive high-throughput study at the protein level is needed to determine the proteins that interact with ERH. In this study, a series of analyses of ERH-binding proteins were carried out to explore the molecular biological mechanism of the ERH protein in tumorigenesis and BC development. Additionally, we overexpressed ERH protein in human T24 cells and then performed coimmunoprecipitation (co-IP) followed by shotgun mass spectrometry (MS), which is a high-throughput assay to characterize protein interactomes (11), to explore the proteins that interact with ERH in human T24 cells.

## 2 Materials and Methods

### 2.1 Ethics Statement

All experimental procedures were approved by the ethics committee of Xuzhou Central Hospital.

### 2.2 Reagents and Devices

D-Hanks solution and PBS were obtained from Shanghai Genechem Technology Co., Ltd. (332 Edison Road, Zhangjiang Hi-Tech Park, Pudong New Area, Shanghai, China). Protein A/G PLUS-Agarose (Sc-2003) was purchased from Santa Cruz Bioengineering (Shanghai) Co., Ltd. Zhangjiang Hi-Tech Park, Pudong, Shanghai, China), and fetal bovine serum (FBS, VS500T) was purchased from Ausbia. Shanghai First Chemical Reagent Co., Ltd. (1317 Jianchuan Road, Minhang District, Shanghai, China). Dimethyl sulfoxide (DMSO, 130701), SDS, Tris, iodoacetamide, NH_4_HCO_3_, formic acid, acetonitrile, H_2_O_2_, Dulbecco’s minimum essential medium (DMEM, 10–013-CVR), paraformaldehyde, RPMI 1640 medium, streptomycin and penicillin. Pentobarbital sodium, dithiothreitol (DTT, 43819–5G), HCOONH_4_ (17843) trifluoroacetate (TFA, T6508), propidium iodide (PI, P4170), and Giemsa staining solution (32884) were obtained from Sigma Company (Building C, 15–18, Front Beach World Trade Center, No. 3, Lane 227, Dongyu Road, Pudong New Area, Shanghai). Anti-GAPDH antibody, protein A/G PLUS-agarose, rabbit anti-human primary antibody (ERH, HPA002567), mouse anti-human primary antibody (EIF2α, L57A5, #2103), donkey anti-mouse IgG H&L (Alexa Fluor^®^ 488, ab150105) and goat anti-rabbit IgG H&L (DyLight^®^ 594, ab96885) secondary antibodies were purchased from Santa Cruz Biotechnology, Inc. (10410 Finnell Street, Dallas, Texas 75220, USA.). A BCA Protein Assay kit (P0010S) and RIPA lysis buffer (strong) (P0013B) were purchased from the Beyotime Institute of Biotechnology (No. 123, Rongle East Road, Songjiang District, Shanghai, China). Phosphotungstic acid (GZ02535), a prestained protein marker (00161543) and an ECL Plus Kit (M3121/1859022) were purchased from Thermo Fisher Scientific Co., Ltd. (Thermo Fisher Scientific, 168 Third Avenue, Waltham, MA, USA, 02451). X-ray film developer powder and fixing powder (P61–04–1) were obtained from the Shanghai Guanlong Photographic Material Factory (No. 221, Jinling East Road, Shanghai, China).

A stabilized power supply (EPS-300), SDS–PAGE protein electrophoresis instrument (VE-180) and the protein transfer equipment (VE-186) were purchased from Shanghai Tianneng Life Science Co., Ltd. (No. 10 Oasis Ring Road, Minhang District, Shanghai). A refrigerated high-speed centrifuge (Fresco 21) was provided by Thermo. An inverted fluorescence microscope (XDS-100, Shanghai Caikang Optical Instrument Co., Ltd.), a microplate reader (M2009PR, Tecan Infinite), a CO_2_ incubator (SANYO, MCO-175), and a fluorescence microscope (IX71, Olympus) were used in this study.

### 2.3 Cells and Cell Culture

Human T24 cells were purchased from Cell Resource Center, Shanghai Institutes for Biological Sciences, and Chinese Academy of Sciences. The cells were cultured in RPMI 1640 medium containing 10% FBS, streptomycin, and penicillin at 37°C in an incubator with 5% CO_2_ (Sanyo, MCO-15A).

### 2.4 ERH Overexpression Lentiviral Vector Construction

In this research, the ERH gene was used as a template to design overexpression primers and primers to amplify ERH gene fragments. The 5’ and 3’ end sequences of the amplified products were consistent with the end sequences of the linearized cloning vector. The recombinant product was transformed into cells and a single clone was PCR-identified to obtain high-purity fragment and used for viral packaging. The cloning site was set at the Age I/Nhe I, GV367 (purchased from Shanghai Genechem Co., Ltd.) was selected as the vector, and the sequence of elements was Ubi-MCS-SV40-EGFP-IRES-puromycin. The ERH gene amplification lentiviral vector was subsequently constructed.

The sequences of the ERH amplification primers were 3’-’ GAGGATCCCCGGGTACCGGCGCCACCATGGACTACAAAGACCATGACGGTGATTATAAAGATCATGACATCGATTACAG-5’ and 3’-CACACATTCCACAGGCTAGCTTATTTCCCAGCCTGTTG GGCCTGC-5’. The two ERH primers used for PCR were 3’-GGGTCAATATGTAATTTTCAGTG-5’ and 3’-CGTCGCCGTCCAGCTCGACCAG-5’. We sequenced the clones and selected clones with the same sequencing results as the target for subsequent experiments.

We then used fluorescently labeled lentivirus with an overexpression vector to infect target cells and promote target gene expression in target cells.

- To prepare the target cells, the cells were cultured in a 37°C, 5% CO_2_ incubator. The culture medium was replaced every 24 hours until the cells reached ~80% confluency prior to subculturing.
- Lentivirus infection: The cells in the logarithmic growth phase were trypsinized to prepare a cell suspension of 3–5×10^4^/m. The inoculation volume of ​​the 6-well plate was approximately 2 ml, the volume of the medium was 1 ml, and the infection medium was replaced with conventional medium after 8–12 hours of infection.
- The cell status and infection efficiency were observed. If the cell status was good without excessive cell death, and the cell infection rate exceeded 70%, downstream experiments were performed. IV. Cells in good condition with sufficient infection efficiency were used for the downstream experiments. We then carried out Western blotting to verify the expression of the ERH gene in the two groups of human T24 cells.

### 2.5 Co-IP and Shotgun Mass Spectrometry (MS) Analysis

For Western blot assays to analyze the overexpression of ERH proteins, cells were washed twice using cold PBS. The BCA approach was used to detect the protein concentration. Proteins (30–50 μg) were resolved by 10% Bis-Tris gradient SDS–PAGE under reducing conditions and transferred onto polyvinylidene fluoride (PVDF) membranes that were blocked with 5% skim milk for 1 hour. The membranes were sequentially incubated with primary antibodies (FLAG, Sigma) followed by an antibody against glyceraldehyde-3-phosphate dehydrogenase (GAPDH) at room temperature for 2 hours. The membranes were then washed with TBST 4 times (8 min each) and incubated with fluorescein-linked secondary antibodies at room temperature for 1.5 hours. Then, the membranes were washed with TBST another 4 times (8 min each), and signals were detected using ECL and X-ray film.

For IP analysis, whole-cell extracts were prepared in NP-40 lysis buffer (50 mM Tris-HCl (pH 7.5), 150 mM NaCl, 5 mM EDTA, 1% NP-40, 0.5% deoxycholate, 0.1% SDS), incubated with antibodies or IgG at 4°C for 2 hours, and then mixed with 100 μl protein A/G agarose overnight at 4°C. Recovered proteins associated with ERH or IgG were resolved by gel electrophoresis. The bands containing proteins that specifically bound to ERH were excised and added to 1 mL of 100 mM NH_4_HCO_3_/30% ACN to remove the color. The supernatant was removed and the samples were freeze-dried. Subsequently, 180 μL of 100 mM NH4HCO3 and 20 μL of 100 mM DTT was added, and the samples were incubated at 56°C for 30 min. The supernatant was removed, and 100 μL ACN was added. The μL ACN was aspirated after 5 min, and 140 μL 100 mM NH_4_HCO_3_ and 60 μL 200 mM IAA was added. The samples were then incubated in the dark for 20 min. The supernatant was removed, and 200 mM NH4HCO3 was added. The samples were subsequently incubated for 15 min. The supernatant was removed, and 100 μL ACN was added. The supernatant was removed after 5 min. The samples were then freeze-dried. Subsequently, 20 μL2.5~10 ng/μL Trypsin solution was added, and the samples were rehydrated in the refrigerator at 4°C for 30 min. Approximately 40 μL of 25 mM NH_4_HCO_3_ solution was added, and the samples were incubated at 37°C for 20 hours. The enzymolysis solution was then aspirated and transferred to a new EP tube, and 100 μL of 60% ACN/0.1% TFA was added to the original tube and subsequently ultrasonicated for 15 min. The solution was aspirated and added to the previous solution, freeze-dried and reconstituted in 0.1% TFA. A C18 cartridge was used to desalt the peptide. After freeze-drying the peptide, 10 μL 0.1% FA was used for the Q-Exactive test.

LC–MS/MS was performed using a Q-Exactive mass spectrometer coupled with an Easy nLC (Thermo Fisher Scientific, MA, USA). The peptide sample was first loaded onto a C18 reversed-phase analytical column (Thermo Scientific, Acclaim PepMap RSLC 50 µm x 15 cm, nano viper, P/N164943) in buffer A (0.1% formic acid in HPLC-grade water) and separated with a linear gradient of buffer B (80% acetonitrile and 0.1% formic acid) with a flow rate of 300 nL/min. A linear chromatographic gradient was achieved with a linear increase in buffer B percentage, which was set up as follows: 6% buffer B for 5 min, 6–28% buffer B for 40 min, 28–38% buffer B for 5 min, 38–100% buffer B for 5 min, and held in 100% buffer B for 5 min.

Subsequently, the peptide was transferred to the Q Exactive mass spectrometer (Thermo Fisher Scientific, MA, USA). The MS analysis was set for 60 min in the positive ion mode. MS data were acquired using a data-dependent top10 method dynamically choosing the most abundant precursor ions from the full scan (350–1800 m/z) for HCD fragmentation. Full scans were acquired at a resolution of 70000 at m/z 200 with an AGC target of 3e6 and a maxIT of 50 ms. MS2 scans were acquired at a resolution of 17500 for HCD spectra at m/z 200 with an AGC target of 2e5 and a maxIT of 45 ms. The scan range was 200–22000 m/z, and the isolation width was 2 m/z. Only ions with a charge state between 2–7 and a minimum intensity of 2e3 were selected for fragmentation. The dynamic exclusion for selected ions was 30 s. The microscan was 1. The normalized collision energy was 27 eV.

### 2.6 Protein Identification and ERH-Interactive Protein List

We used Proteome Discoverer 2.2 (Thermo Fisher Scientific) for protein identification in the raw data and the Mascot 2.6 app for the database search. The UniProt database (UniProt_HomoSapiens_20394_20210127) was used for the functional annotation and classification of identified proteins. The search parameters included peptides with a maximum of 2 missed cleavages, a precursor mass tolerance of 10 ppm, and 0.05 Da tolerance for MS2 fragments. The fixed modification was carbamidomethyl, and variable modifications were oxidation and acetylation. Proteins were considered positively identified if the peptide score of a specific peptide reached the significance threshold FDR = 0.01. The positive protein list of the ERH NC group was extracted from that of the ERH OE (overexpression) group to obtain a list of proteins that can interact with the ERH protein. In order to reduce the problem of interference, we performed a negative control experiment, and compared the ERH overexpression group with ERH normal group. Among the obtained binding proteins, we subtracted the binding protein list of the NC group from the binding protein list of the ERH-OE group, to exclude the non-specific binding proteins. Next, we conducted a series of comprehensive analyses on this list to identify the molecular biological role of ERH protein in human T24 cells. Finally, an ERH-IPL was obtained and bioinformatics analyses was performed.

### 2.7 Bioinformatics Analysis of ERH-IPL

In this study, various network bioinformatics analysis tools were used, such as GO and KEGG analysis, Metascape network, PPI-MCODE of Cytoscape, TCGA database, UALCAN, and GeneMINIA.

#### 2.7.1 GO, KEGG and Protein–Protein Interaction Analyses of Related Genes by Metascape

The GO (Gene Ontology) database is divided into three parts: cellular component (CC), molecular function (MF), and biological process (BP). KEGG (Kyoto Encyclopedia of Genes and Genomes, https://www.kegg.jp/) is a bioinformatics resource for understanding the functions and utilities of biological systems, such as cells, organisms and ecosystems, for molecular-level information. Metascape (http://metascape.org) is a comprehensive and detailed bioinformatics analysis website that includes STRING, PPI, WiKiPathways, GO, and KEGG analysis. The database of this website is updated monthly, and the last updated date was 2021–08–01 as of the time of our data analysis.

#### 2.7.2 Analysis of PPI by STRING and GeneMANIA

We referred to STRING network tools (https://string-db.org) to analyze the PPIs of the ERH-IPL and visualized it with Cytoscape (version 3.8.2). The molecular complex detection (MCODE) functions of Metascape and Cytoscape were performed to analyze the most connected clusters. We then used GeneMANIA network tool (http://genemania.org/) to analyze the PPIs in the STRING results to find the key gene list and the most important interactive protein. As we obtained the list of key genes, we referred to UALCAN, a network TCGA database analysis tool (http://ualcan.path.uab.edu/index.html) to obtain the BC prognostic survival curves of the key genes.

### 2.8 Immunofluorescence Colocalization Assay

Immunofluorescence colocalization assay was used to explore the co-expression of ERH and the most important interacting protein in human T24 cells. Briefly, T24 cells were divided into ERH NC and ERH KD groups. A labeled rabbit anti-human EIF2α antibody was used, followed by red fluorescently labeled goat anti-rabbit IgG. ERH was labeled with mouse anti-human antibody and then localized with green fluorescently labeled donkey anti-mouse IgG. Nuclear staining was performed with DAPI staining. Cells at the logarithmic phase were used to prepare a 2 × 10^4^/ml single-cell suspension, seeded in a 96-well plate, and cultured in a carbon dioxide incubator for 1–3 days. The cells were fixed with 4% paraformaldehyde and rinsed with PBS. Then, 0.2% Triton was added, and the cells were rinsed with PBS for 30 min. The cells were incubated with primary and secondary antibodies, and fluorescence photography was performed with a fluorescence microscope.

### 2.9 Protein Docking Prediction

Using the Discovery Studio 4.0 software, we analyzed the protein molecular structure of the two proteins and used the ZDOCK technology of the rigid protein docking algorithm based on the fast Fourier transform related technology to make a prediction. A simulated docking image of the two proteins was obtained along with docking scores and specific binding sites (hydrogen bonds). The fast Fourier transform correlation technique in the algorithm was used to search the translational and rotational spaces of the protein system, and these binding configurations were scored using an energy scoring function.

### 2.10 Statistical Analysis

Statistical analyses were performed using SAS 9.43 (SAS Institute Inc., Cary, NC, USA). The statistical significance of differences in continuous data (mean ± SD) was estimated using Student’s t-test. For all the analyses, a p-value <0.05 indicated statistical difference (*), and a p-value <0.01 indicated a significantly statistical difference (**).

## 3 Results

### 3.1 Establishment of the ERH OE Bladder Cancer T24 Cell Line and ERH-IPL

**Figure 1:**
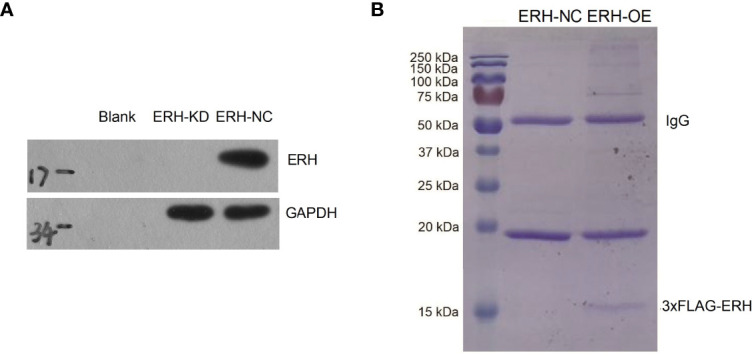
Fluorescence-labeled lentivirus was used for infection. The expression of the reporter gene was observed with a fluorescence microscope 72 hours after infection. The infection rate was over 70%. **(A)** Western blot assay results showed ERH overexpressed in the ERH OE group at the protein level. **(B)** Coomassie blue staining of affinity-purified ERH and ERH-associated proteins. MW indicates molecular weight.

The ERH OE bladder cancer T24 cell line was established by lentiviral transfection. The transfection efficiency exceeded 70% (**Figure 1A**). The Western blot results showed that ERH expression was increased at the protein level in the ERH OE group compared to the control group, and the difference was significant (**Figure 1B**). To identify proteins that interact with ERH, we isolated ERH-interacting proteins using 3×FLAG-tagged ERH (**Figure 1C**). We obtained a list of 205 proteins that can directly or indirectly interact with ERH protein by mass spectrometry (**Supplementary Table 1**).

### 3.2 Biological Function Enrichment: Metascape GO and KEGG Analysis

**Figure 2:**
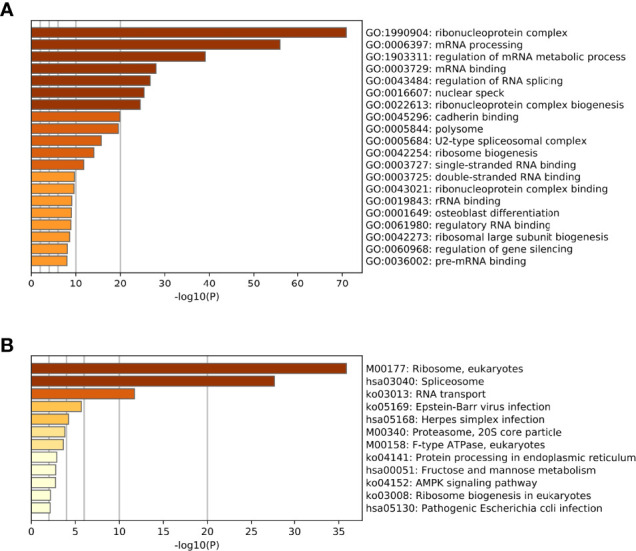
**(A)** The biological function enrichment results of the GO analysis. The GO database is divided into three modules: Cellular Components, Molecular Functions and Biological Processes. Columns are sorted by P-value, which is consistent with **Table 1**. **(B)** The biological function enrichment results of the KEGG analysis. Columns are sorted by P-value, which is consistent with **Table 2**.

Referring to the Metascape website, we obtained the biological function enrichment results of GO (**Table 1**) and KEGG (**Table 2**) related to ERH-IPL which interacted with ERH protein in human T24 cells (**Figure 2**).

**Table 1:** GO biological function enrichment.

GO	Cat.	Description	Count	%	Log10 (P)	Log10 (q)
GO:1990904	CC	ribonucleoprotein complex	79	39.11	-70.90	-66.55
GO:0006397	BP	mRNA processing	62	30.69	-55.87	-52.13
GO:1903311	BP	regulation of mRNA metabolic process	42	20.79	-39.07	-35.87
GO:0003729	MF	mRNA binding	40	19.80	-28.02	-25.16
GO:0043484	BP	regulation of RNA splicing	25	12.38	-26.70	-23.87
GO:0016607	CC	nuclear speck	34	16.83	-25.34	-22.54
GO:0022613	BP	ribonucleoprotein complex biogenesis	35	17.33	-24.46	-21.70
GO:0045296	MF	cadherin binding	27	13.37	-19.89	-17.24
GO:0005844	CC	polysome	16	7.92	-19.55	-16.94
GO:0005684	CC	U2-type spliceosomal complex	15	7.43	-15.71	-13.22
GO:0042254	BP	ribosome biogenesis	21	10.40	-14.04	-11.60
GO:0003727	MF	single-stranded RNA binding	12	5.94	-11.77	-9.38
GO:0003725	MF	double-stranded RNA binding	10	4.95	-9.68	-7.34
GO:0043021	MF	ribonucleoprotein complex binding	12	5.94	-9.58	-7.25
GO:0019843	MF	rRNA binding	9	4.46	-9.08	-6.77
GO:0001649	BP	osteoblast differentiation	14	6.93	-9.01	-6.70
GO:0061980	MF	regulatory RNA binding	8	3.96	-8.90	-6.60
GO:0042273	BP	ribosomal large subunit biogenesis	9	4.46	-8.62	-6.32
GO:0060968	BP	regulation of gene silencing	11	5.45	-8.10	-5.82
GO:0036002	MF	pre-mRNA binding	8	3.96	-8.01	-5.74

Cat, Category; CC, cellular components; MF, molecular functions; BP, biological processes.

**Table 2:** KEGG biological function enrichment.

KEGG	Cat.	Description	Count	%	Log10(P)	Log10(q)
M00177	SC	Ribosome, eukaryotes	27	13.37	-35.86	-33.18
hsa03040	Pathway	Spliceosome	25	12.38	-27.61	-25.42
ko03013	Pathway	RNA transport	15	7.43	-11.71	-9.58
ko05169	Pathway	Epstein–Barr virus infection	10	4.95	-5.68	-3.88
hsa05168	Pathway	Herpes simplex infection	8	3.96	-4.22	-2.58
M00340	SC	Proteasome, 20S core particle	3	1.49	-3.81	-2.21
M00158	SC	F-type ATPase, eukaryotes	3	1.49	-3.64	-2.08
ko04141	Pathway	Protein processing in endoplasmic reticulum	6	2.97	-2.89	-1.37
hsa00051	Pathway	Fructose and mannose metabolism	3	1.49	-2.77	-1.30
ko04152	Pathway	AMPK signaling pathway	5	2.48	-2.75	-1.30
ko03008	Pathway	Ribosome biogenesis in eukaryotes	4	1.98	-2.17	-0.79
hsa05130	Pathway	Pathogenic Escherichia coli infection	3	1.49	-2.14	-0.78

Cat, Category; SC, Structural Complexes.

### 3.3 PPI-MCODE Analysis and Key Gene Identification

**Figure 3:**
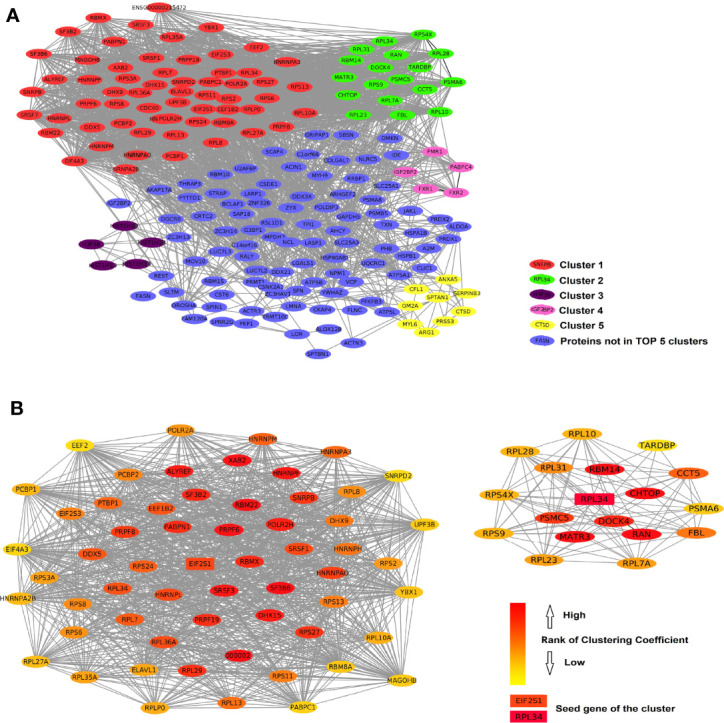
**(A)** The protein–protein interactions of ERH-IPL. Red shows the most closely linked cluster 1 (score 39.00 with 70 nodes), green shows cluster 2 (score 9.88 with 18 nodes), purple shows cluster 3 (score 5.00 with 5 nodes), pink shows cluster 4 (score 4.00 with 5 nodes), yellow shows cluster 5 (score 3.00 with 9 nodes), and blue shows the genes not in the top five clusters. **(B)** shows the network of clusters 1 and 2, ranked by clustering coefficient, indicating that they interact with each other. EIF2S1 and RPL34 were considered the seed genes and are marked as key genes.

We analyzed the PPIs by Metascape and obtained a list of MCODEs. The main cluster of proteins interacting with ERH consisted of those with MCODEs related to “ribonucleoprotein complex”, “ATPase activity”, “nuclear speck”, and “translation factor activity, RNA binding”. The interactive data were visualized by Cytoscape software. A function of “MCODE” was used to find the tightest clusters. The criteria of MCODE to obtain the cluster were degree cutoff: 2, node score cutoff: 0.2, K-core: 2, max depth: 100. We obtained 7 clusters, and the top 5 are shown in **Figure 3A**. We analyzed the first and second clusters to rank the proteins by clustering coefficient (**Figure 3B**). With a review of previous literature and analysis of network resources, we finally identified 16 genes that may be the key genes related to the ERH gene in human T24 cells.

**Figure 4:**
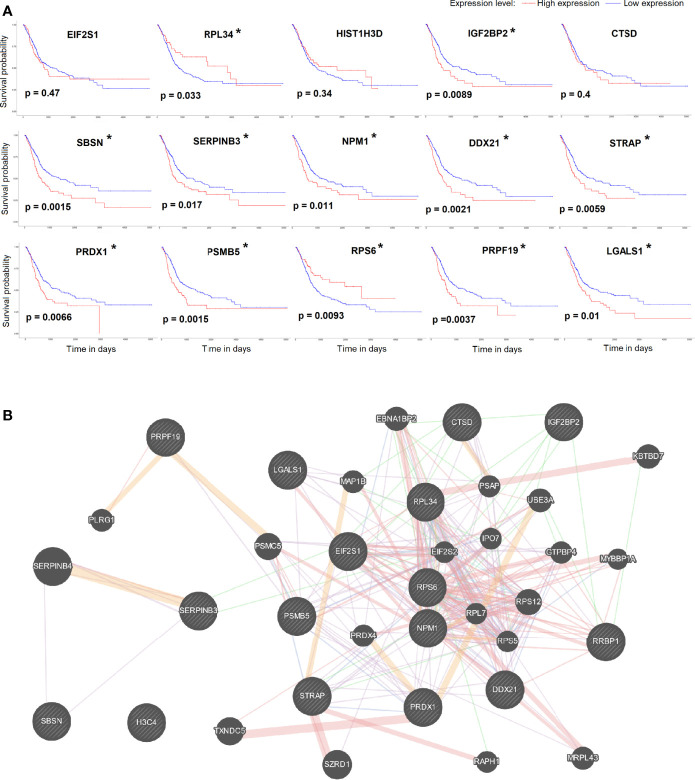
**(A)** The survival curve of key genes analyzed by the UALCAN network TCGA database analysis tool. P<0.05 was considered significant (*). **(B)** The pathways and co-expression status of the key genes.

Using the UALCAN network TCGA database analysis tool, we obtained bladder urothelial carcinoma prognostic survival curves using the 15 key genes. The results showed that the expression levels of 12/15 of the key genes can predict the prognosis of bladder urothelial carcinoma (**Figure 4A**). We further analyzed the pathways and co-expression status of these 15 key genes using the GeneMINIA website (**Figure 4B**). As EIF2S1 is the key gene in the first cluster, we set EIF2S1 as the most important interactive protein and performed an immunofluorescence colocalization assay.

### 3.4 ERH Interacts With EIF2α in Human T24 Cells

**Figure 5:**
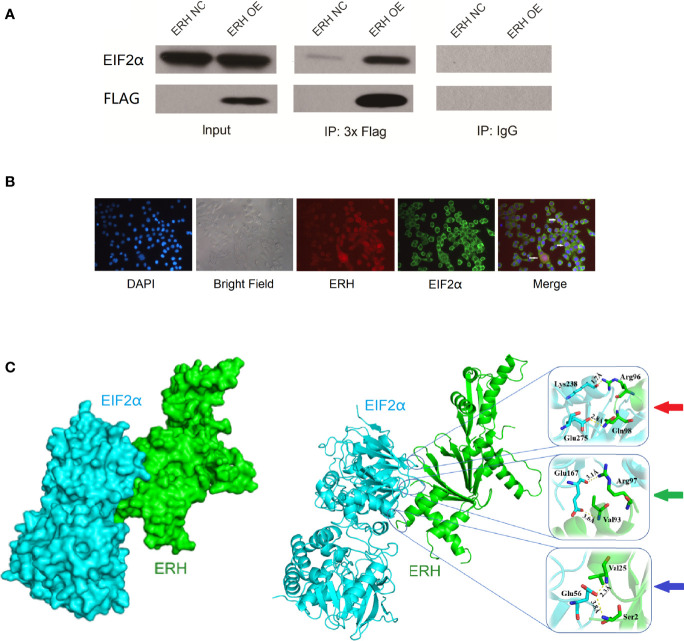
**(A)** IP assay showing EIF2α detected in the IP-3×Flag samples from both the NC and OE groups. EIF2α was significantly increased in the OE group compared to in the NC group. EIF2α was not detected in the IP-IgG samples from either the NC or OE groups, indicating that 3xFlag-ERH can immunoprecipitate EIF2α; therefore, ERH and EIF2α may directly interact. **(B)** Immunofluorescence colocalization assay. Red fluorescence indicates ERH expression. Green fluorescence shows EIF2α expression. The “Merge” shows that ERH and EIF2α were co-expressed in human T24 cells. **(C)** The left image shows the chimeric structure of the molecular docking, and the right image shows the hydrogen bonding at the junction. Red arrow: amino acids Lys238 and Glu275 in EIF2α protein form two hydrogen bonds with amino acids Arg96 and Gln98 in ERH protein, and the interaction distances are 1.7 Å and 2.8 Å, respectively. Green arrow: amino acid Glu167 in EIF2α protein forms two hydrogen bonds with amino acids Arg97 and Val93 in ERH protein, and the interaction distances are 3.1 Å and 3.6 Å, respectively. Blue arrow: amino acid Glu56 in EIF2α protein forms two hydrogen bonds with amino acid Val25 and Ser2 in ERH protein, and the interaction distances are 2.3 Å and 3.8 Å, respectively.

We identified EIF2α as an ERH-interacting protein by MS analysis. We tested the interaction between ERH and EIF2α by an IP assay and assessed the ability of 3×Flagged ERH and EIF2α to coimmunoprecipitate. The results showed that ERH and EIF2α were co-expressed and interacted in human T24 cells (**Figure 5A**).

As confirmed by immunofluorescence colocalization assay, ERH and EIF2α (the α-subunit of eukaryotic initiation factor 2) were co-expressed in some states of T24 cells (**Figure 5B**).

The fraction of docking with ERH protein with EIF2α protein is -88.969. There are 6 hydrogen bonds on the predicted bonding surface of the two proteins (**Figure 5C**).

### 3.5 ERH Regulates the EIF2α-ATF4/CHOP Pathway

**Figure 6:**
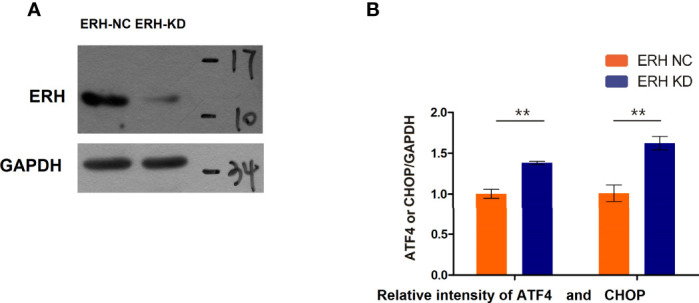
**(A)** Western blot analysis. The ERH was knocked down in the ERH KD group. **(B)** The EIF2α-ATF4/CHOP pathway-related mRNAs were upregulated in the ERH KD group. ** Significant difference between groups (P<0.01)

qPCR analysis revealed that ATF4 and CHOP expression levels were increased in human T24 cells following ERH KD. The expression levels of ATF4 and CHOP in the ERH KD group were significantly increased compared to those in the ERH NC group (**Figure 6**).

## 4 Discussion

This study was designed to determine the proteins that interact with the ERH protein in human T24 cells by co-IP and MS assays. Additionally, we explore the protein interacting role of the ERH protein by bioinformatics network analysis. To confirm the results of our network bioinformatics analysis, we selected one of the analytic results for verification, and the verification results were consistent with the bioinformatics analysis.

Co-IP and MS are commonly used methods to detect protein–protein interactions in cells (11, 12). We overexpressed the ERH gene and obtained 205 proteins that might directly or indirectly interact with ERH in human T24 cells.

GO biological function enrichment results showed that ERH interactive proteins were related to “ribonucleoprotein complex”, “mRNA processing” and “regulation of mRNA metabolic process” as the top three categories, ranked by p-value. As demonstrated by Xie *et al.* in 2019 (13), the ERH protein can form a dimer to provide a conserved link between RNA-binding proteins and RNA-processing effectors. Another study (7) revealed that in a novel protein complex PETISCO (PID-3, ERH-2, TOFU-6, and IFE-3 small RNA complex), the ERH protein can interact directly with PID-1 and TOST-1 to affect 21 U RNA biogenesis. These studies indicated that the ERH protein is closely related to mRNA processing and RNA metabolic processes, consistent with our GO biological function enrichment analysis.

The KEGG biological function enrichment results showed that the ERH interactive proteins were related to “Ribosome, eukaryotes”, “Pathway Spliceosome” and “RNA transport” as the top three categories, ranked by p-value. ERH, as a molecular partner of PDIP46/SKAR, can interact with ribosomes, as shown by a study conducted by Pogge *et al.* (14). ERH interacts with multiple RNA processing complexes, including many splicing regulators, such as BCLAF1, THRAP3, C1QBP, CHTOP, and POLDIP3 (15). Their study also revealed that the ERH protein interacts with DGCR8, DROSHA and FAM208B, that are related to microRNA biogenesis. Another study (16) conducted in 2012 revealed that ERH can combine with mRNA splicing of the mitotic motor protein CENP-E and is involved in mRNA splicing and mitosis. Drakouli *et al.* found in 2017 that (17) ERH interacts directly with the C-terminal Arg-Gly-rich region of SAFB1/2 in the nucleus, which is involved in mRNA splicing. ERH has been found to be involved in RNA transport in other studies (18, 19). Therefore, our KEGG biological function enrichment results were consistent with previous studies.

The PPI-MCODE biological function enrichment results show that ERH interactive proteins were related to “ribonucleoprotein complex”, “nuclear-transcribed mRNA catabolic process, nonsense-mediated decay” and “mRNA splicing, *via* spliceosome” as the top three categories, ranked by p-value. The PPI-MCODE biological function enrichment analysis results were consistent with the KEGG analysis results (**Table 3**). Next, we selected one of the key proteins of the PPI-MCODE biological function enrichment clusters to verify the accuracy of the network biological information analysis.

**Table 3:** PPI-MCODE biological function enrichment.

MCODE	GO	Description	Log10(P)
MCODE_1	GO:1990904	ribonucleoprotein complex	-76.2
MCODE_1	GO:0000184	nuclear-transcribed mRNA catabolic process, nonsense-mediated decay	-54.9
MCODE_1	GO:0000398	mRNA splicing, via spliceosome	-52.4
MCODE_2	GO:0016887	ATPase activity	-3.6
MCODE_2	GO:0072594	establishment of protein localization to organelle	-3.3
MCODE_3	GO:0016607	nuclear speck	-8.4
MCODE_3	GO:1990904	ribonucleoprotein complex	-5.2
MCODE_3	GO:0000398	mRNA splicing, via spliceosome	-4.3
MCODE_4	GO:0008135	translation factor activity, RNA binding	-6.6
MCODE_4	GO:0090079	translation regulator activity, nucleic acid binding	-6.3
MCODE_4	GO:0045182	translation regulator activity	-5.9

As confirmed by immunofluorescence colocalization assays, ERH and EIF2α were co-expressed in human T24 cells in certain circumstances. We predicted the binding sites of the two proteins using rigid docking of protein molecules. The expression levels of ATF4 and CHOP, which are downstream of EIF2α, were increased in human T24 cells following ERH KD. The expression of ATF4 and CHOP in the ERH KD group was significantly increased compared to that in the ERH NC group. The verification of the interacting protein EIF2α indicated that co-IP/MS followed by biological network information analysis was feasible. The study of molecular interactions at the proteomics level using this method has been widely applied for decades in molecular biology research.

The EIF2α-ATF4/CHOP pathway is regulated by ER stress-related ferroptosis in cancer cells (20–22). Phosphorylation of EIF2α can induce the translation of ATF4 and, consequently, CHOP expression (23). It has been reported that the EIF2α-ATF4/CHOP pathway is regulated by NF-κB (22). As we have reported in a previous article (6), the NF-κB networks are upstream regulators of the ERH gene. The results of this experiment suggest that the downstream regulatory pathway of the ERH gene might be the EIF2α-ATF4/CHOP pathway; thus, NF-κB may affect the EIF2α-ATF4/CHOP pathway by regulating ERH gene expression.

## 5 Conclusion

In summary, we confirmed that ERH protein affects “ribonucleoprotein complex”, “ATPase activity”, “nuclear speck”, and “translation factor activity, RNA binding” in human T24 cells. The ERH protein interacts with EIF2α and regulates the EIF2α-ATF4/CHOP signaling pathway in T24 cells. Therefore, targeting ERH could be considered as a new strategy for bladder cancer treatment.

## Data Availability Statement

The datasets presented in this study can be found in online repositories. The names of the repository/repositories and accession number(s) can be found in the article/**Supplementary Material**.

## Ethics Statement

The studies involving human participants were reviewed and approved by Xuzhou Central Hospital Ethics Committee. Written informed consent for participation was not required for this study in accordance with the national legislation and the institutional requirements.

## Author Contributions

Conceptualization, KP, YD, Z-SC and C-hH. Methodology, KP, YD, LH, Z-dS, Z-gZ, BC, Y-yM, HX, DP, Z-sC and C-hH. Writing – original draft preparation, KP, YD, HF. Writing – review & editing, Z-sC and C-hH. Supervision, Z-sC and C-hH.

## Funding

Chinese National Natural Science Fund (82004110). Jiangsu Province key research and development program (BE2020758, BE2019637). Jiangsu Province, the medical innovation team (CXTDA2017048). Jiangsu Province Traditional Chinese Medicine Science and Technology Development Plan Project (MS2021051). Scientific research project of Jiangsu Provincial Health Commission (H2018051). The Key Projects of Xuzhou Science and Technology Plan (KC19075, KC21263). Xuzhou clinical medicine expert team project (2018TD004). Xuzhou Medical University Excellent Talent Fund Project (XYFY2020014, XYFY2020026).

## Conflict of Interest

The authors declare that the research was conducted in the absence of any commercial or financial relationships that could be construed as a potential conflict of interest.

## Publisher’s Note

All claims expressed in this article are solely those of the authors and do not necessarily represent those of their affiliated organizations, or those of the publisher, the editors and the reviewers. Any product that may be evaluated in this article, or claim that may be made by its manufacturer, is not guaranteed or endorsed by the publisher.

## Abbreviations

CC, cellular component; BC, bladder cancer; BP, biological process; co-IP, coimmunoprecipitation; ERH, enhancers of rudimentary homolog; ERH-IPL, ERH-interactive protein list; GO, gene ontology; KD, knocked-down; KEGG, Kyoto Encyclopedia of Genes and Genomes; MCODE, molecular complex detection; MS, mass spectrometry; MF, molecular function; NC, normal cell; OE, over-expression; PETISCO, PID-3, ERH-2, TOFU-6, and IFE-3 small RNA complex; PPI, protein–protein interaction; TCGA, The Cancer Genome Atlas; UALCAN, the university of Alabama at Birmingham Cancer data analysis portal.
