# Supplementary figures and images for: ERH Interacts With EIF2α and Regulates the EIF2α/ATF4/CHOP Pathway in Bladder Cancer Cells
Source: Front Oncol. 2022 Jun 14;12:871687. doi: 10.3389/fonc.2022.871687 (PMC9239699; doi:10.3389/fonc.2022.871687)

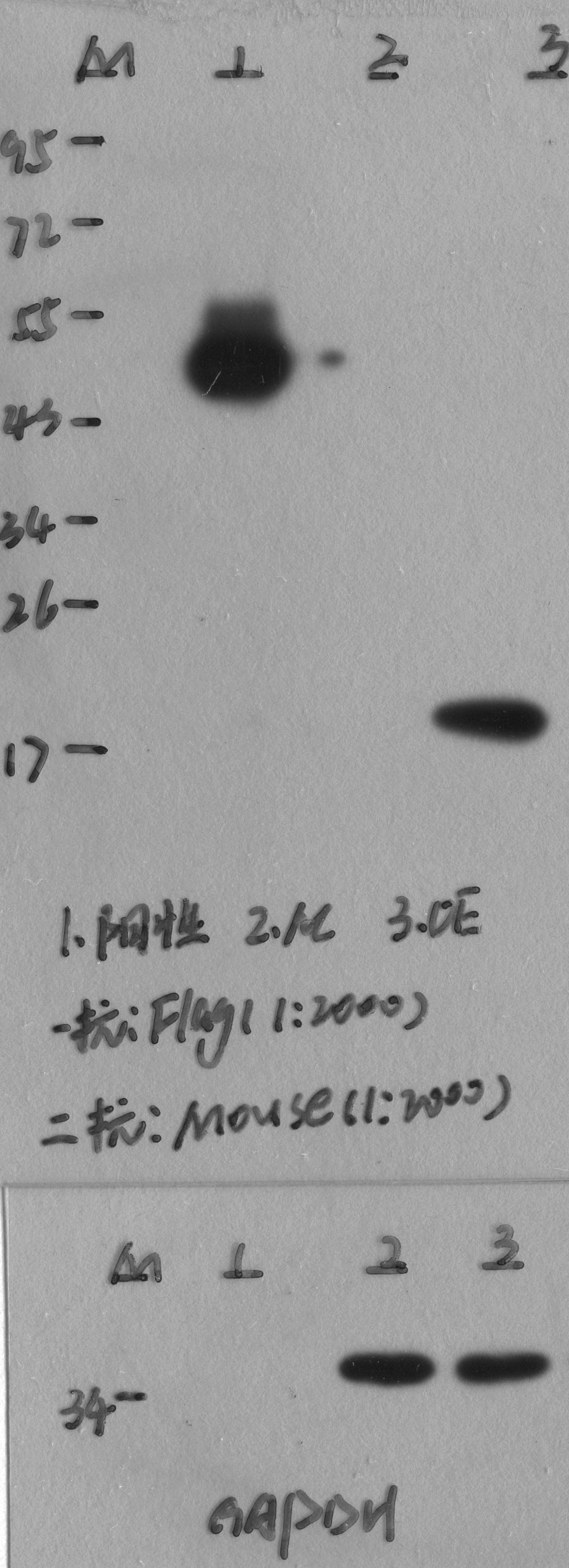

Supplement: Supplementary file 1 [file Image_1.jpeg]

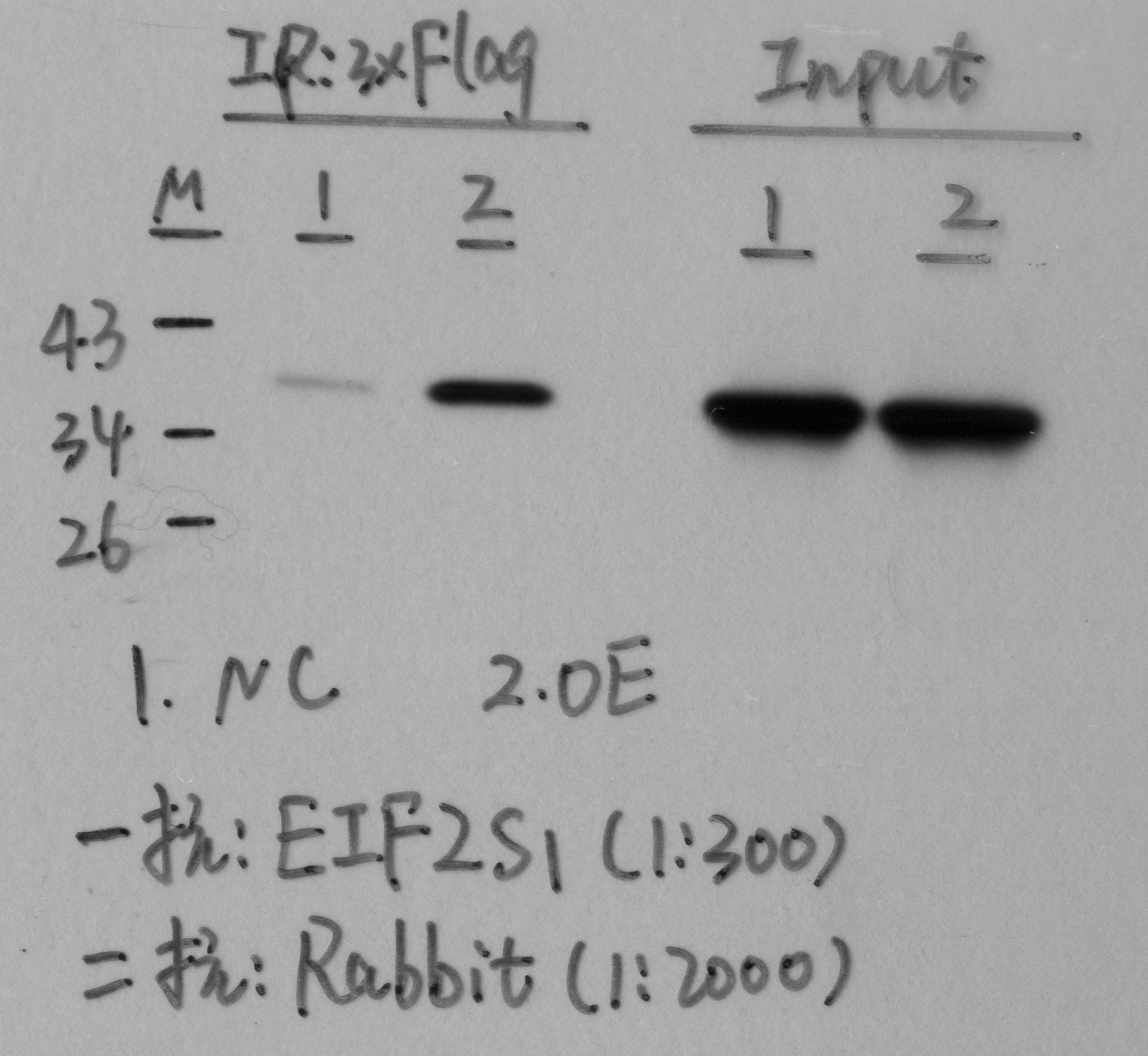

Supplement: Supplementary file 2 [file Image_2.jpeg]

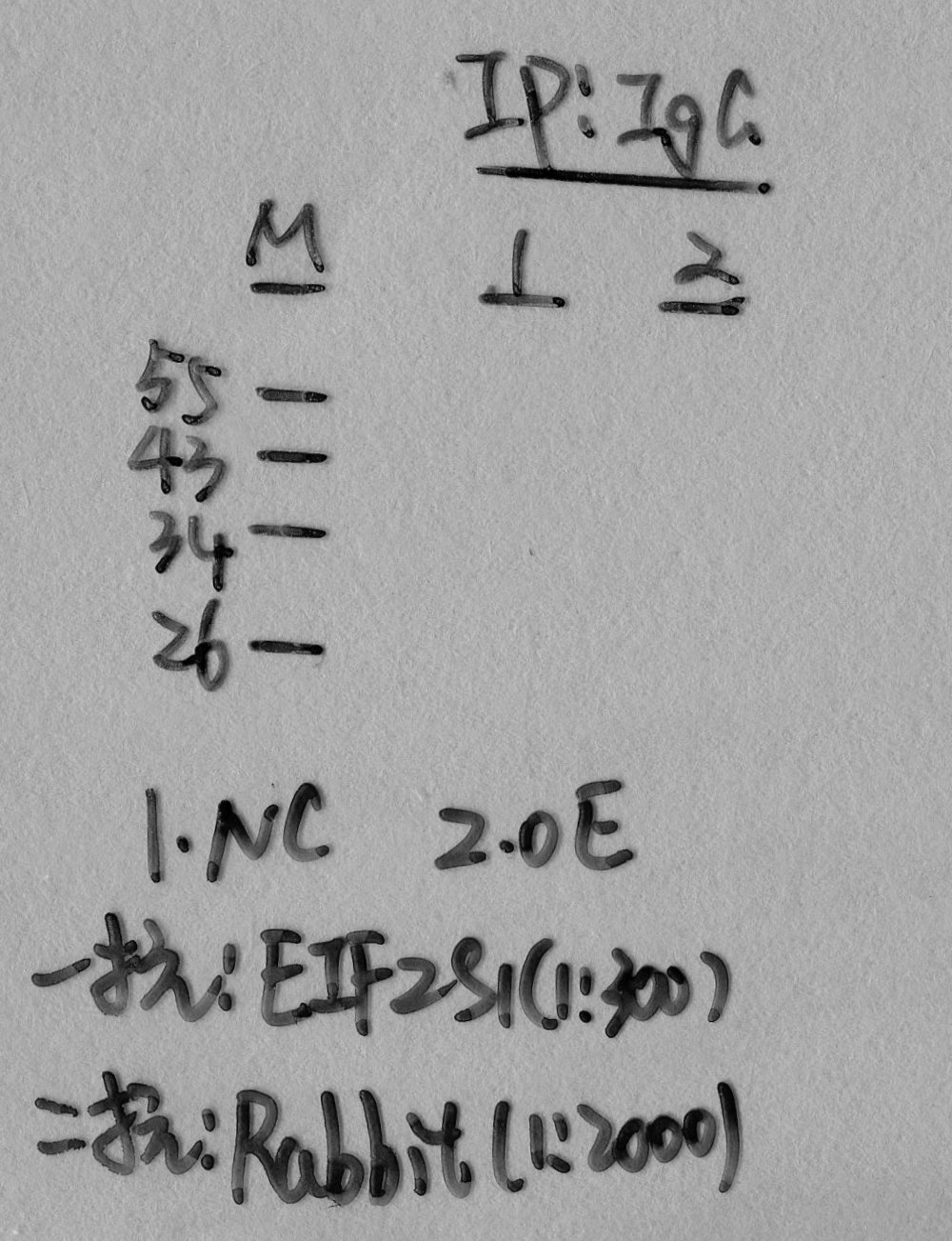

Supplement: Supplementary file 3 [file Image_3.jpeg]

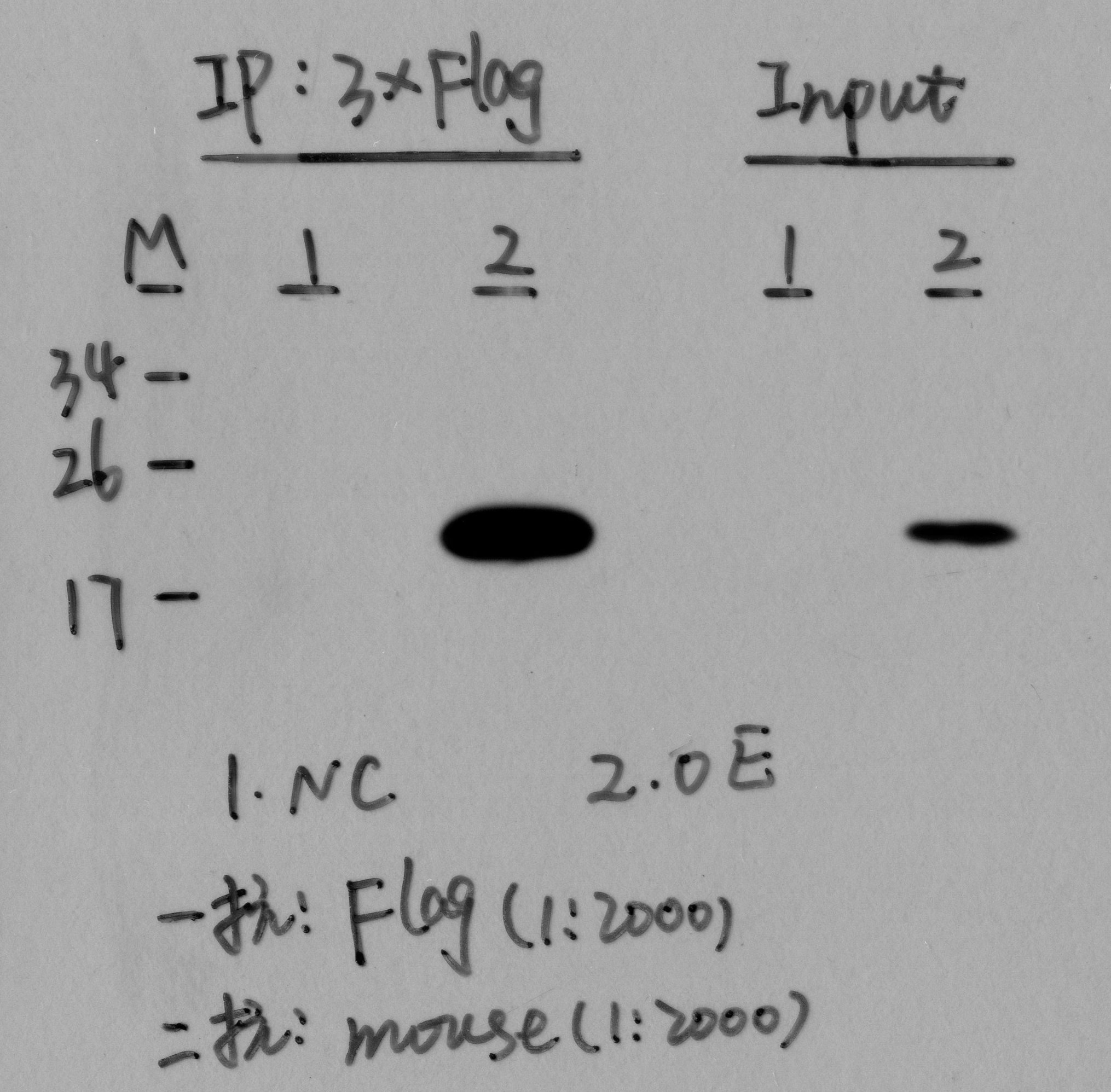

Supplement: Supplementary file 4 [file Image_4.jpeg]

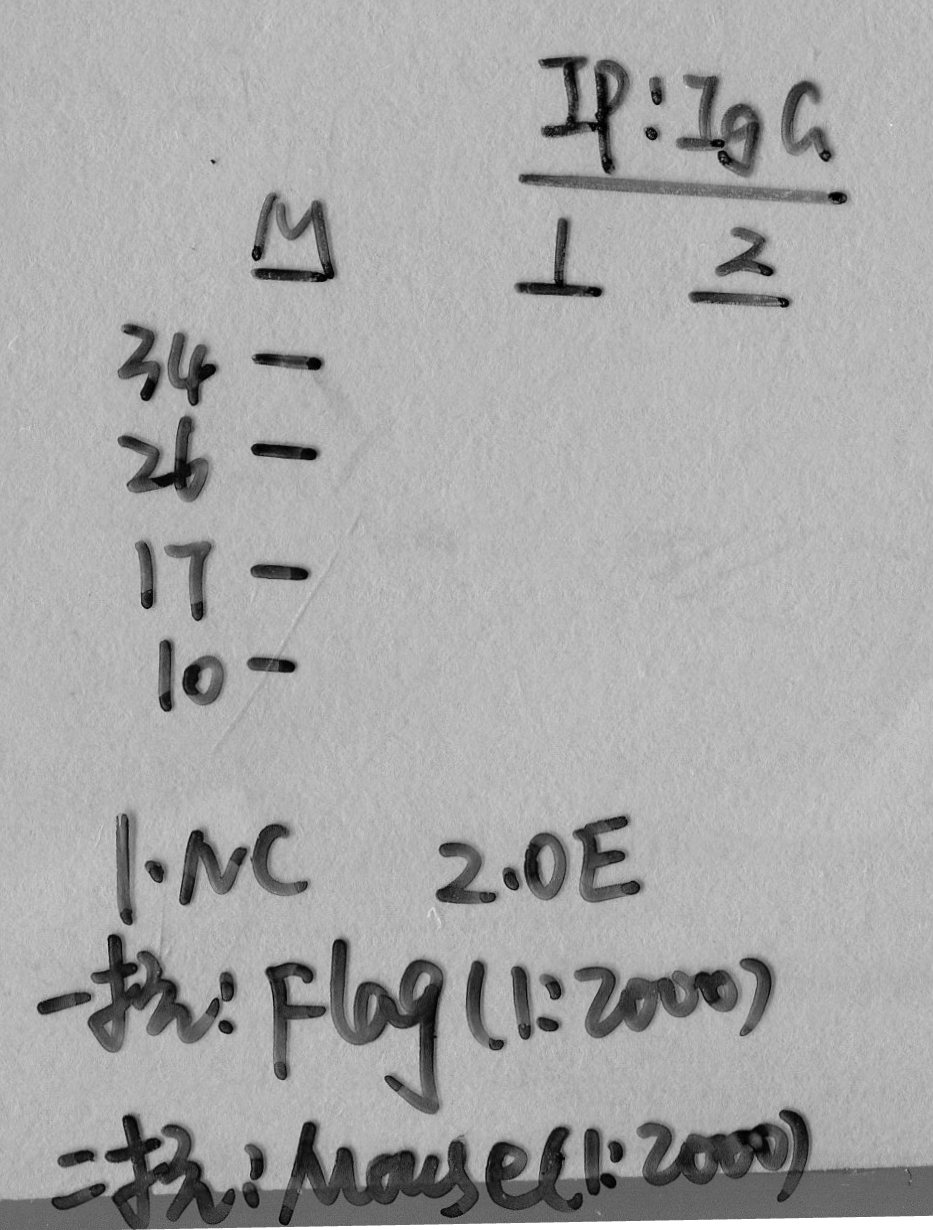

Supplement: Supplementary file 5 [file Image_5.jpeg]
